# Evaluation of Surface Cleaning Procedures in Terms of Gas Sensing Properties of Spray-Deposited CNT Film: Thermal- and O_2_ Plasma Treatments

**DOI:** 10.3390/s17010073

**Published:** 2016-12-30

**Authors:** Joon Hyub Kim, Min-Jung Song, Ki Beom Kim, Joon-Hyung Jin, Nam Ki Min

**Affiliations:** 1 Department of Electro-Mechanical Systems Engineering, Korea University, 2511 Sejong-ro, Sejong City 339-770, Korea; kim4539@korea.ac.kr (J.H.K.); kkb5009@korea.ac.kr (K.B.K.); 2 College of Liberal Art & Interdisciplinary Studies, Kyonggi University, 154-42 Gwanggyosan-ro, Yeongtong-gu, Suwon-si, Gyeonggi-do 443-760, Korea; mjsong@kyonggi.ac.kr; 3 National Institute for Nanomaterial Technology, POSTECH 77, Cheongam-ro, Nam-gu, Phohang, Gyeongbuk 37673, Korea; 4 Department of Chemical Engineering, Kyonggi University, 154-42 Gwanggyosan-ro, Yeongtong-gu, Suwon-si, Gyeonggi-do 443-760, Korea

**Keywords:** ammonia gas sensor, oxygen plasma cleaning, single-walled carbon nanotube, spray coating, thermal cleaning

## Abstract

The effect of cleaning the surface of single-walled carbon nanotube (SWNT) networks by thermal and the O_2_ plasma treatments is presented in terms of NH_3_ gas sensing characteristics. The goal of this work is to determine the relationship between the physicochemical properties of the cleaned surface (including the chemical composition, crystal structure, hydrophilicity, and impurity content) and the sensitivity of the SWNT network films to NH_3_ gas. The SWNT networks are spray-deposited on pre-patterned Pt electrodes, and are further functionalized by heating on a programmable hot plate or by O_2_ plasma treatment in a laboratory-prepared plasma chamber. Cyclic voltammetry was employed to semi-quantitatively evaluate each surface state of various plasma-treated SWNT-based electrodes. The results show that O_2_ plasma treatment can more effectively modify the SWNT network surface than thermal cleaning, and can provide a better conductive network surface due to the larger number of carbonyl/carboxyl groups, enabling a faster electron transfer rate, even though both the thermal cleaning and the O_2_ plasma cleaning methods can eliminate the organic solvent residues from the network surface. The NH_3_ sensors based on the O_2_ plasma-treated SWNT network exhibit higher sensitivity, shorter response time, and better recovery of the initial resistance than those prepared employing the thermally-cleaned SWNT networks.

## 1. Introduction

Since 2000, considerable progress has been made in the development of carbon nanotube (CNT)-based chemical gas sensors, both theoretically and experimentally. The high surface-to-volume ratio of CNTs can catalyze the chemisorption or physisorption between CNT surfaces and various gases or vapors. This eventually enhances the sensitivity of the CNT substrate to any target materials, even at room temperature. Indeed, numerous articles focusing on different aspects of CNT-based chemical sensors have been published, including a summary of the progress made thus far in gas sensing performance and potential applications in other fields. Although the working mechanism or the exact chemistry that occurs on the CNT surface upon exposure to a target gas has yet to be confirmed [1,2,3,4], CNTs are undoubtedly promising materials for various sensing applications.

In spite of the considerable potential of CNTs as gas sensing materials, a crucial issue that needs to be fully considered is the method of cleaning the individual CNT or CNT networks before use. Contaminants and impurities that are inevitably introduced to the CNTs during the synthesis and the following purification procedure can seriously affect the working mechanism of the CNT-based sensors in their detection of external target materials [5]. Actually, pristine CNTs are chemically inactive and easily aggregated by van der Waals force; the attraction energy between CNTs is about 500 eV∙μm^−1^ [6,7]. More recent advances in CNT chemistry have presented the successful dissolution and dispersion of CNTs into various solvents [8,9]. However, organic solvents such as *N,N*-dimethylformamide (DMF), *N*-methylpyrrolidone, and *o*-dichlorobenzene (DCB) that are frequently used to prepare a homogeneously-dispersed CNT solution commonly form an outermost organic thin layer on the CNT-based sensing material. These electrically nonconductive residual contaminants should be carefully removed to avoid affecting the sensing performance of factors such as sensitivity, electron transfer rate, and the reproducibility of the CNT gas sensors. It has been reported that the surface states of CNTs play a very important role in the response of CNT-based sensing devices such as chemiresistors [10], field-effect transistors [11,12,13], and biosensors [14,15,16].

In this study, we present for the first time results obtained from a systematic approach to evaluate the O_2_ plasma treatment and thermal treatment for cleaning a single-walled carbon nanotube (SWNT) network and the resulting influence on NH_3_ gas sensing performance. The electrodes of the patterned SWNT networks are spray-coated, and subsequently modified with thermal annealing and O_2_ plasma treatment at various conditions. The removal efficiency of the organic residuals in the SWNT network is determined by observing the contact angle and chlorine percentage in X-ray photoelectron spectroscopy (XPS) analysis. The surface characteristics and electrochemical properties of the SWNT network are analyzed by Raman spectroscopy, cyclic voltammetry, and square-wave voltammetry. Finally, the influence of the thermal and plasma cleaning procedures on the NH_3_ gas sensing properties of the spray-deposited SWNT networks is investigated.

## 2. Materials and Methods

### 2.1. Chemicals and Materials

Commercially available SWNTs (≈70% purity) were obtained from Hanwha Nanotech Co. (Incheon, Korea). DCB (C_6_H_4_Cl_2_) was purchased from Sigma-Aldrich (St. Louis, MO, USA) and was used as a solvent to prepare a stable SWNT-dispersed solution. Three milligrams of the SWNTs were tip-sonicated in 150 mL of DCB for 20 min, and centrifuged at 4 °C and 20,000 rpm for 20 min. The supernatant is the SWNT-dispersed DCB solution.

### 2.2. Preparation of the Modified SWNT Electrodes

A 20 nm-thick Ta under-layer was deposited on a glass substrate, followed by the deposition of a 200 nm-thick Pt thin film with radio-frequency (RF) sputtering. The Ta thin film enhances the adhesive strength between Pt and the glass substrate. The previously prepared SWNT-dispersed DCB solution is then spray-coated directly onto the Pt layered glass substrate. The approximate amount of the loaded SWNT is 1 mL∙cm^−2^. In order to remove any residual organic solvents from the electrode surface, the SWNT-patterned Pt electrode is either heat-cured or O_2_ plasma treated. For thermal cleaning, the SWNT electrode is placed on a programmable hot plate at various temperatures (100–400 °C) for 1 h with a ramp rate of 4 °C∙min^−1^. The heat-cured SWNT is referred to as t-SWNT. For the O_2_ plasma cleaning, the experimental condition is optimized in terms of the plasma power (10 and 20 W) and the duration (5–30 s), where the O_2_ flow rate and the substrate temperature are 30 sccm and 25 °C, respectively. The resulting O_2_ plasma-treated SWNT is denoted as p-SWNT. The optimized t-SWNT or p-SWNT films were deposited on the interdigitated electrodes to prepare two different NH_3_ gas sensors.

### 2.3. Characterization and Gas Sensing Measurement

The morphology and structure of the heat or O_2_ plasma-cured SWNTs were investigated using scanning electron microscopy (SEM; S-4300, Hitachi Ltd., Tokyo, Japan). To approximately identify the removal of organic materials and the surface restructuring on the modified film, contact angle measurements were taken before and after the cleaning of the SWNT networks using a commercially-available contact angle system (Phoenix-10, SEO Co., Seoul, Korea). Surface characterization of the modified electrode was analyzed using Raman (LabRAM, Horiba KOREA, Bucheon, Korea) and XPS (SIGMA PROBE, ThermoVG, London, UK). The electrochemical measurements were performed using 263A Potentiostat/Galvanostat (Princeton Applied Research, Oak Ridge, TN, USA) at room temperature. To record the square wave voltammograms, the following instrumental parameters were used: step potential = 1 mV; square-wave amplitude = 2 mV; frequency = 10 kHz; and scan rate = 50 mV∙s^−1^. The NH_3_ sensing performance of the SWNT-based gas sensors was evaluated in a laboratory-prepared gas sensing system composed of a furnace, the gas control system (control of NH_3_ gas concentration in the detection chamber can be accomplished by adjusting the ratio of gas flow rate of NH_3_ to that of N_2_), a Keithley 236 source meter, a PC, and gas cylinders of NH_3_ and N_2_ at an operating temperature of 250 °C.

## 3. Results and Discussion

**Figure 1:**
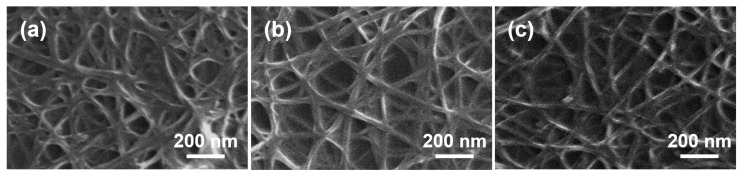
SEM images of the modified single-walled carbon nanotube (SWNT) bundles: (**a**) before cleaning; (**b**) thermal cleaning; and (**c**) plasma cleaning.

**Figure 2:**
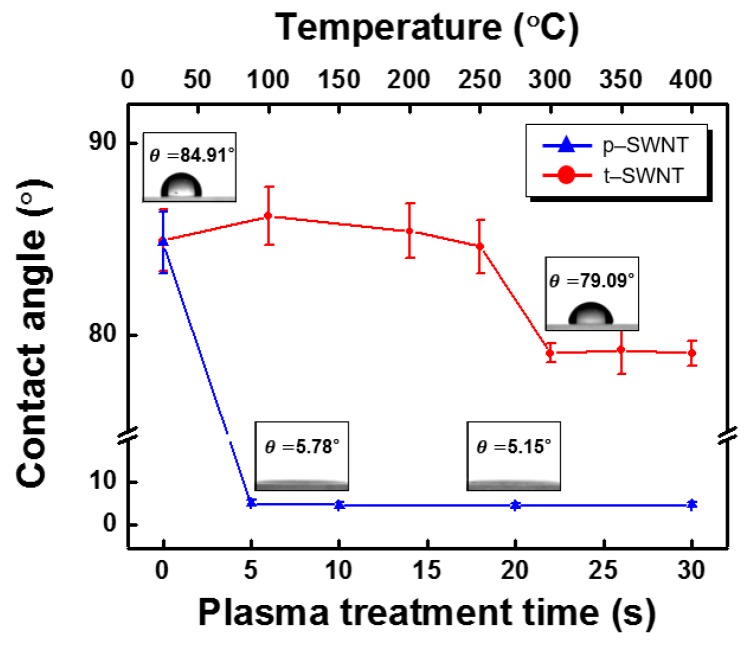
Static contact angle data for the heat-cured SWNT (t-SWNT) and O_2_ plasma-treated SWNT (p-SWNT) at various conditions. Note that the p-SWNT shows much smaller standard deviation of the observed contact angle than the t-SWNT does.

Figure 1 shows SEM images of the variously prepared SWNT bundles: (a) before cleaning; (b) after thermal cleaning; and (c) after plasma cleaning. As shown in Figure 1a, the pristine SWNT was susceptible to aggregation, and each individual SWNT was coated by the unwanted, DCB-involved residual sonopolymers [17,18,19,20]. Both cleaning processes can effectively remove the residual organic surfactants to provide a naked SWNT bundle. However, the cleaned surface state strongly depends on the cleaning process employed. The contact angle of the water droplet on the DCB-coated glass substrate which is not treated with heat was about 62.51°, owing to the hydrophobic nature of DCB. The contact angle remained almost unchanged with the DCB-coated glass substrates annealed at a temperature below 250 °C, but the angle decreased significantly with the glass substrates that were heat treated at a temperature higher than 250 °C and up to 400 °C. This means that the DCB molecules are thermally decomposed at a temperature above 250 °C, eventually enabling the hydrophilic property of the glass substrate to be recovered. Figure 2 shows the static contact angle data for the t-SWNT and p-SWNT prepared at various conditions. The pristine SWNT surface is more hydrophobic than the DCB-coated glass substrate, and the observed contact angle is about 84.91°, because both the SWNT and DCB molecules are hydrophobic. In the case of the t-SWNT annealed at temperatures higher than 300 °C, the contact angle decreased by only about 6.9% with the removal of the DCB molecules. Conversely, the p-SWNT showed a dramatic contact angle decrease by 93.2% or more. This is attributed to the removal of organic residuals and to the hydrophilic modification of the SWNT’s surface; i.e., O_2_ plasma chemically and more effectively oxidizes the hydrophobic SWNT surface than the thermal treatment, so the O_2_ plasma-treated surface becomes more hydrophilic, since the oxygen-containing functional groups (mainly carboxyl) are more effectively introduced to the SWNT surface.

**Figure 3:**
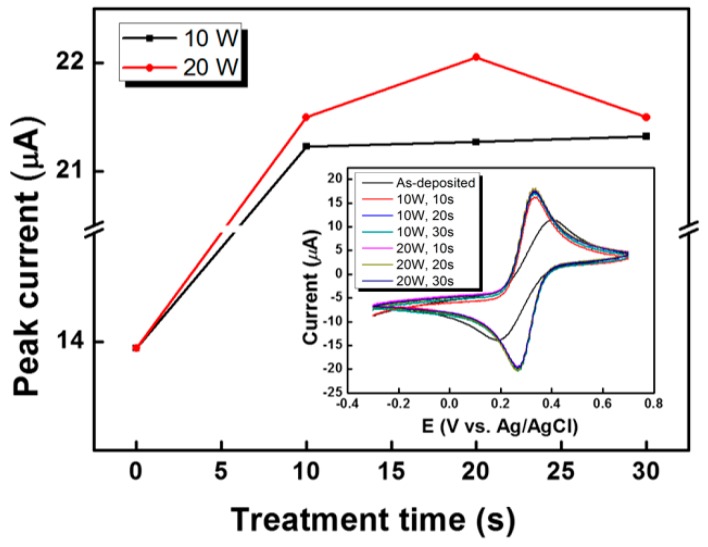
Peak current (i_p_) of the p-SWNT at various conditions. Detailed experimental conditions for the cyclic voltammograms are as follows: redox species = 10 mM K_3_[Fe(CN)_6_], electrolyte = 3 M KCl solution, and scan rate = 100 mV∙s^−1^.

The plasma treatment condition for preparing p-SWNT is optimized mainly in terms of the applied plasma power and the plasma treatment time. The peak current (i_p_) obtained from the cyclic voltammograms of the variously prepared p-SWNT electrodes in a 3 M KCl solution containing 10 mM K_3_[Fe(CN)_6_] at 100 mV∙s^−1^ (inset of Figure 3) is plotted as a function of the plasma treatment time, as shown in Figure 3. The results indicate that the i_p_ of the p-SWNT electrodes treated with O_2_ plasma at 20 W were larger than those obtained at 10 W; the larger i_p_ means an increase in the conductivity of the p-SWNT electrode prepared at 20 W power O_2_ plasma. The enhanced conductivity is probably due to the larger number of carboxyl or carbonyl substituents on the p-SWNT surface. However, a long plasma treatment time can reduce the conductivity of the p-SWNT electrode, giving a decreased i_p_, which results from a decreased concentration of the oxygen-containing chemical functional groups on the SWNT surface due to the over-etched SWNTs [21]. Finally, the optimal condition for p-SWNT is obtained at 20 W for 20 s.

**Figure 4:**
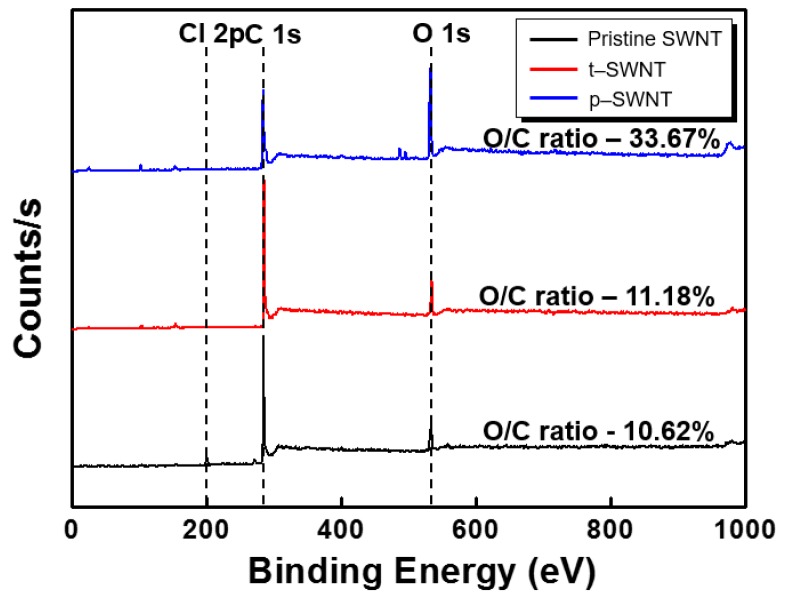
Changes of the core level spectra of SWNT by thermal- and plasma-cleaning treatments.

**Figure 5:**
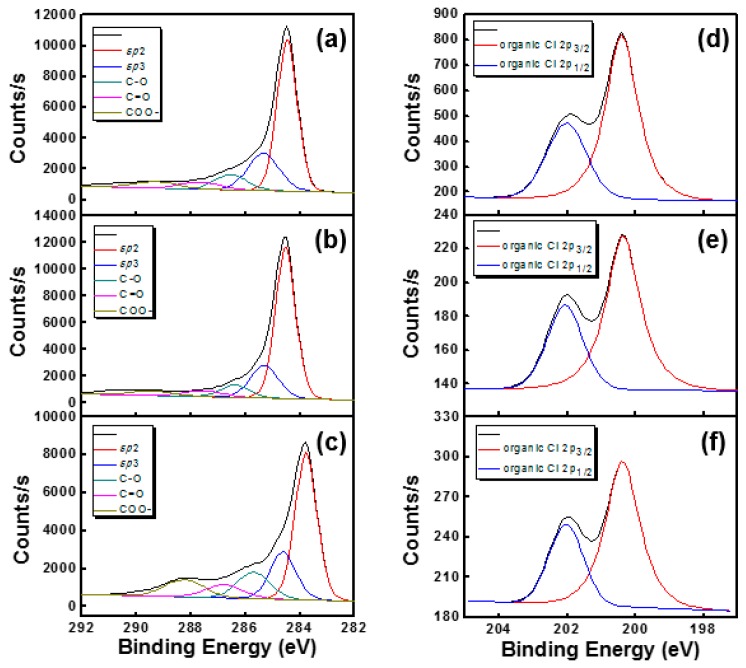
X-ray photoelectron spectroscopy (XPS) peak deconvolution of the C 1s for (**a**) pristine SWNT; (**b**) t-SWNT and (**c**) p-SWNT and Cl 2p peaks for (**d**) pristine SWNT; (**e**) t-SWNT and (**f**) p-SWNT.

Figure 4 shows the difference in the core level spectra of SWNTs obtained by thermal- and plasma-cleaning treatments. The XPS spectrum shows distinct carbon, oxygen, and chlorine peaks, representing the major constituents of the SWNT surface. Here, the peak of chlorine (200.34–200.40 eV) is associated with the organic chlorocarbons from the residual DCB molecules. The XPS peak deconvolution of the C ls and Cl 2p peaks for the modified SWNTs is illustrated in Figure 5. As shown in Figure 5a–c, the C 1s spectra for the modified SWNTs have five characteristic peaks, including the elemental carbon atoms (sp^2^ and sp^3^) and the oxidized carbon atoms (C–O, C=O, and –COO). The concentrations of C, O, and Cl for all the samples were estimated, and the corresponding results are summarized in Table 1. Compared with the pristine SWNT, the ratio of oxygen-bound carbons to carbon atoms ([CO_x_]/[C]) increases in both the heat treatment and O_2_ plasma treatment. Especially, a larger number of oxidized species on the SWNTs is observed after the O_2_ plasma treatment. The p-SWNT shows a decrease in sp^2^ bonding and an increase in sp^3^ bonding, revealing that the sp^2^ hybridization of SWNTs is altered to sp^3^ hybridization, indicating that the characteristics of SWNT (such as electrical conductivity and mechanical strength) gradually disappear [21,22]. The chlorine Cl 2p core level spectra are depicted in Figure 5d–f. The t-SWNT and p-SWNT electrodes induce a less-intense Cl 2p component with a relatively low binding energy of 200.34–200.36 eV. This could be attributed to a decrease in the concentration of the residual DCB sonopolymers.

**Figure 6:**
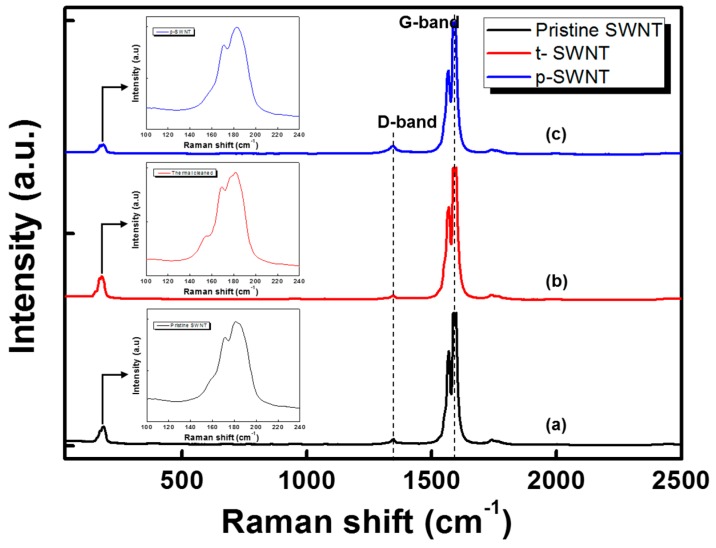
Raman spectra for the modified SWNTs: (**a**) pristine SWNT; (**b**) t-SWNT; and (**c**) p-SWNT.

Figure 6 shows the Raman spectra of the modified SWNT bundles: (a) pristine SWNT; (b) t-SWNT; and (c) p-SWNT. The intensity ratios of the D-band to the G-band (I_D_/I_G_) for the modified SWNT bundles were estimated in order to evaluate the degree of structural deformation [18]. Generally, the G and D bands are characterized as ordered carbon in a tangential mode and as disordered sp^2^ carbon, respectively. The I_D_/I_G_ value of pristine SWNT was about 0.0518, which means that pristine SWNT exhibited some structural damage owing to acid treatment. It is known that most commercially available SWNTs are purified by acid and heat treatments during synthesis in order to improve purity. For the p-SWNT sample, the I_D_/I_G_ was approximately 0.0717, which is greater than the pristine SWNT, attributed to the defect generation or chemical functionalization on the end edge and even on the sidewalls of the SWNT surface by the O_2_ plasma treatment. In contrast, the I_D_/I_G_ of t-SWNT was about 0.0451, which is smaller than that of the pristine SWNT, indicating that the thermal treatment of the SWNT does not generate significant defects on the SWNT surface. The Raman spectra of the modified SWNTs in the spectral region of the radial breathing mode (RBM) at 170–183 cm^−1^ are demonstrated in the insets of Figure 6. The diameter of SWNT can be estimated using Equation (1).

(1) $$\omega =\frac{c_{1}}{d}\;+{\;c}_{2}$$

where ω is the RBM frequency; d is the diameter; c_1_ and c_2_ (as the experimentally derived parameters) are 223.5 cm^−1^ and 12.5 cm^−1^, respectively, for SWNT bundles. For all samples, two resolved Radial Breathing Mode peaks were observed, and their corresponding diameters are summarized in Table 2. It has been reported that the radical species react more selectively with SWNTs of a smaller diameter [18]. Based on this, it can be concluded that p-SWNT shows excellent reactivity in comparison to pristine SWNT and t-SWNT.

**Figure 7:**
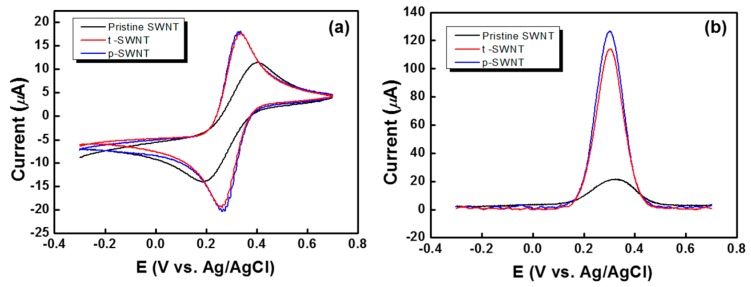
Electrochemical properties of the modified electrodes in a 3 M KCl solution containing 10 mM K_3_[Fe(CN)_6_] at 100 mV∙s^−1^: (**a**) cyclic voltammograms and (**b**) square-wave voltammograms. Note that the p-SWNT shows the smallest peak potential separation and the biggest i_p_. This indicates the best surface condition of the p-SWNT electrode. Note that the modified electrodes are prepared under optimal treatment conditions.

As shown in Figure 7a, the cyclic voltammograms of all electrodes in a 3 M KCl solution containing 10 mM K_3_[Fe(CN)_6_] at 100 mV∙s^−1^ exhibit well-defined and quasi-reversible redox reactions, indicating that effective electron transfer occurs between the electrode and the electrolyte containing the redox active species. The pristine SWNT electrode showed a relatively lower peak current (i_p_) and a wider peak-to-peak separation potential (ΔE_p_) than the t-SWNT and p-SWNT electrodes. The p-SWNT exhibited the smallest ΔE_p_ (about 0.0648 V, as shown in Figure 7a), and the highest i_p_ (ca. 127.01 μA, as shown in Figure 7b) than the other SWNTs. The magnitude of the electrochemical response for these electrodes is in the following order: p-SWNT > t-SWNT > pristine SWNT (Figure 7b). The excellent electrochemical properties of the p-SWNT electrode due to a sufficient amount of carbonyl/carboxyl substituents result in enhanced conductivity and electron transfer acceleration, which play important roles in electronic devices.

**Figure 8:**
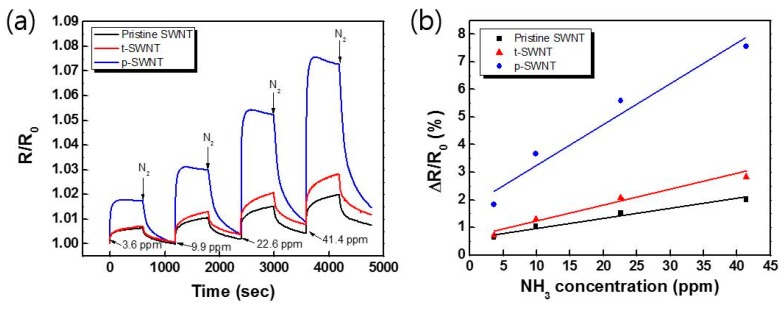
(**a**) Dynamic response of the modified SWNT sensors to different concentrations of NH_3_ at 250 °C and (**b**) their calibration curves. Note that the p-SWNT-based sensor reaches a steady state with a slight overshoot shortly after exposure to NH_3_ gas, while the t-SWNT-based and pristine SWNT-based sensors are not yet fully saturated.

Figure 8 shows the dynamic response of the modified SWNT-based sensors at various NH_3_ concentrations. The sensing response is estimated using Equation (2):

(2) $$\mathrm{Response}=\frac{R_{g}-R_{0}}{R_{0}}\times 100\;\left(\%\right)$$

where R_g_ and R_0_ are the electrical resistances before and after exposure to NH_3_, respectively. The response time and the recovery time are defined as the times to reach 90% of total change in electrical resistance upon exposure to target gas and to return to 10% of the original resistance in N_2_ gas after the test gas is released, respectively. Among the three samples, the p-SWNTs sensor exhibited the highest sensitivity and the fastest response time (Figure 8), because the gas sensing performance depends mostly on the concentration of oxygen-containing functional groups and on the defect sites on the SWNT surface. Moreover, the pristine and t-SWNTs sensors showed incomplete recovery of their initial resistance for all concentrations. This might be attributed to a buildup of chemisorbed NH_3_ on the SWNT surface, resulting in irreversible change in the resistance [23]. The sensing performances of the modified SWNTs sensors are listed in Table 3.

## 4. Conclusions

We demonstrated an effective strategy to remove the residual DCB from SWNTs, and showed the influence of surface cleaning procedures on the sensing performance of a SWNT-based NH_3_ gas sensor. Thermal- and O_2_ plasma-cleaning treatments were introduced to remove DCB from the SWNTs, and the surface characteristics and electrochemical properties of the cleaned SWNTs were analyzed. The smaller contact angle and lower chlorine percentage after cleaning verified the removal of the DCB from the SWNTs. Although both the O_2_ plasma and thermal treatments exhibited similar DCB removal, the electrochemical property and reactivity to NH_3_ gas of the p-SWNTs were superior to those of the t-SWNTs, and this was attributed to the smaller diameter of p-SWNTs and larger number of carboxyl/carbonyl substituents on the p-SWNT surface. More importantly, the p-SWNT-based gas sensor showed more enhanced sensing performance to detect NH_3_ gas, such as higher response variations and shorter response/recovery times than the pristine- and t-SWNT-based sensors. While thermal cleaning of the SWNT is to remove organic adsorbents from the nanotube surface, plasma cleaning includes cleaning and functionalization of the SWNT at the same time. Therefore, O_2_ plasma treatment is a smart cleaning technique and can be widely applied to various SWNT-based electrodes or devices.

## Figures and Tables

**Table 1:** XPS results of the modified SWNTs.

Sample	sp^2^		sp^3^		C–O		C=O		–COO		Cl 2p	
	BE (eV)	%	BE (eV)	%	BE (eV)	%	BE (eV)	%	BE (eV)	%	BE (eV)	%
Pristine SWNT	284.47	69.05	285.33	16.79	286.53	6.70	287.68	3.17	289.29	2.89	200.40	3.14
t-SWNT	284.53	71.86	285.30	15.51	286.38	5.81	287.55	2.78	289.46	2.29	200.36	0.47
p-SWNT	284.52	57.85	285.34	18.64	286.48	10.31	287.57	5.14	288.89	6.68	200.34	0.47

**Table 2:** Radial Breathing Mode (RBM) peaks of the modified SWNTs.

Sample	Peak 1 (cm^−1^)	d_1_ (nm)	Peak 2 (cm^−1^)	d_2_ (nm)
Pristine SWNT	170.79	1.4120	180.90	1.3272
t-SWNT	169.10	1.4272	181.46	1.3228
p-SWNT	170.79	1.4120	182.58	1.3141

**Table 3:** Sensing performances of the modified SWNTs.

Sample	Sensitivity (%/ppm)	Response Time (s)	Recovery Time (s)	Lower Detection Limit (ppm)
Pristine SWNT	0.0348	430	-	~0.3
t-SWNT	0.0545	424	-	~0.3
p-SWNT	0.145	58	1066	~0.1
